# Evaluating high-resolution computed tomography derived 3-D joint space metrics of the metacarpophalangeal joints between rheumatoid arthritis and age- and sex-matched control participants

**DOI:** 10.3389/fmed.2024.1387532

**Published:** 2024-05-09

**Authors:** Justin J. Tse, Dani Contreras, Peter Salat, Claire E. H. Barber, Glen S. Hazlewood, Cheryl Barnabe, Chris Penney, Ahmed Ibrahem, Dianne Mosher, Sarah L. Manske

**Affiliations:** ^1^McCaig Institute for Bone and Joint Health, Cumming School of Medicine, University of Calgary, Calgary, AB, Canada; ^2^Department of Radiology, Cumming School of Medicine, University of Calgary, Calgary, AB, Canada; ^3^Department of Community Health Sciences, Cumming School of Medicine, University of Calgary, Calgary, AB, Canada; ^4^Division of Rheumatology, Cumming School of Medicine, University of Calgary, Calgary, AB, Canada

**Keywords:** rheumatoid arthritis, high resolution peripheral quantitative computed tomography, joint space narrowing, metacarpophalangeal joints, joint space metrics

## Abstract

**Introduction:**

Rheumatoid arthritis (RA) is commonly characterized by joint space narrowing. High-resolution peripheral quantitative computed tomography (HR-pQCT) provides unparalleled *in vivo* visualization and quantification of joint space in extremity joints commonly affected by RA, such as the 2nd and 3rd metacarpophalangeal joints. However, age, sex, and obesity can also influence joint space narrowing. Thus, this study aimed to determine whether HR-pQCT joint space metrics could distinguish between RA patients and controls, and determine the effects of age, sex and body mass index (BMI) on these joint space metrics.

**Methods:**

HR-pQCT joint space metrics (volume, width, standard deviation of width, maximum/minimum width, and asymmetry) were acquired from RA patients and age-and sex-matched healthy control participants 2nd and 3rd MCP joints. Joint health and functionality were assessed with ultrasound (i.e., effusion and inflammation), hand function tests, and questionnaires.

**Results:**

HR-pQCT-derived 3D joint space metrics were not significantly different between RA and control groups (*p* > 0.05), despite significant differences in inflammation and joint function (*p* < 0.05). Joint space volume, mean joint space width (JSW), maximum JSW, minimum JSW were larger in males than females (*p* < 0.05), while maximum JSW decreased with age. No significant association between joint space metrics and BMI were found.

**Conclusion:**

HR-pQCT did not detect group level differences between RA and age-and sex-matched controls. Further research is necessary to determine whether this is due to a true lack of group level differences due to well-controlled RA, or the inability of HR-pQCT to detect a difference.

## 1 Introduction

Rheumatoid arthritis (RA) is a disease characterized by inflammation-associated deterioration of joint surfaces that can include a loss of cartilage, a reduction in joint space, and eventual bone-on-bone contact. Symptomatically, this can result in loss of gross (e.g., walking, sitting, standing, etc.) and fine (e.g., writing, grabbing objects, etc.) motor skills, leading to an overall decrease in quality-of-life (QOL) (1–3). For RA, the most affected joints are those within the hands, specifically, the metacarpophalangeal (4) joints (MCP). However, apart from rheumatic diseases, decreases in MCP joint functionality may also be attributed to naturally occurring factors.

Age, sex, and obesity have been shown to negatively impact joint space narrowing and disease progression in hand osteoarthritis (OA). The continual wear and tear of cartilage with increasing age can contribute to a cartilage thinning, resulting in joint space narrowing (5–7). In addition to known sex-based joint size differences (8–10), the inverse relationship between obesity and immune response is known to affect OA disease progression (11, 12). While different hand joints are typically affected in RA (MCP joints) compared with OA (distal interphalangeal and proximal interphalangeal joints), MCP joint changes may be influenced by age, sex, and obesity. However, visualizing and quantifying joint changes within small joints with standard X-ray methods can be challenging.

High-resolution peripheral quantitative computed tomography (HR-pQCT) is an X-ray based imaging technique that can provide unparalleled 3D *in vivo* images of the MCP joints, allowing for accurate and precise quantitative 3D derived joint space metrics. Recently, the Study group for x-trEme Computed Tomography in Rheumatoid Arthritis (13) proposed a consensus method to characterize joint space including metrics to assess mean width, maximum width, minimum width, standard deviation of width, asymmetry and volume (13). This fully automated method provided accurate scan-rescan reproducibility, with reported scan-rescan precision errors ranging from 2.3% (mean width) to 13.3% (asymmetry) (13). Several studies have utilized these metrics to assess disease course and treatment effects in RA (14, 15). However, limited sample sizes (16) have restricted the exploration of whether HR-pQCT joint space metrics differ with age, sex, and obesity.

In this study, we aimed to determine the sensitivity of HR-pQCT-derived joint space metrics for the differentiation between the 2^nd^ and 3^rd^ MCP joints of RA and controls, as a function of age, sex, and obesity. Furthermore, joint health and functionality were evaluated via ultrasound (i.e., effusion and inflammation) and physical tasks (i.e., Jebsen Taylor Hand Function Test), respectively – facilitating the comparison of (a) joint space metrics with function of functional outcomes, and (b) joint function and inflammation between groups.

## 2 Materials and methods

### 2.1 Participants

Eighty (80) participants (*N* = 40 RA and *N* = 40 controls) were recruited for this study, which was approved by the Conjoint Health Research Ethics Board at the University of Calgary (REB19-0387). All participants were > 18 years of age, free of cognitive and physical impairments, and were not pregnant nor planning a pregnancy. RA participants were recruited from the Rheum4U database, an ongoing study that is exploring the quality of care in rheumatoid arthritis patients within Calgary, AB, Canada (17). Through this database, RA participants were identified and recruited if (1) they met the American College of Rheumatology/European League Against Rheumatism 2010 classification criteria for RA (18) and (2) had a disease duration ≥6 months. RA participants were excluded if they had any history of a prior MCP injury, replacement, or complete joint space loss in both hands. For the age- (± 2 years) and sex-matched controls, these participants were recruited via word-of-mouth. Control participants were included if they had no diagnosis of any hand joint problems or inflammatory arthritis, no current pain or swelling within the hands, and had not sought medical attention for any hand injuries within the past 12 months. After providing written informed consent, physical characteristics (sex, age, height, and weight), patient reported outcomes (questionnaires), and imaging data were collected.

### 2.2 HR-pQCT acquisition and processing

High-resolution peripheral quantitative computed tomography (HR-pQCT, XtremeCT II, Scanco Medical, Brüttisellen, Switzerland) scans of the most affected hand were acquired with three 1 cm stacks, with 25% overlap, to encompass the 2nd and 3rd MCP joints (MCP2 and MCP3, respectively) (19). Each stack was acquired at 68 keV, 1,470 𝜇A, 43 ms dwell time, and with an isotropic 60.7 𝜇m voxel size. Images were scored for motion (20), and were omitted from further analysis if motion scores >3.

Joint space width analysis was performed using the consensus-based algorithm previously published by (13) using the manufacturer’s software (IPL v5.42, Scanco Medical) (13). From the automatically generated contours of the metacarpal and phalanx bones, joint space volume (JSV), mean width (JSW), standard deviation of width (JSW_SD_), maximum joint space width (JSW_MAX_), minimum joint space width (JSW_MIN_), and width asymmetry (JSW_AS_ = JSW_MAX_/JSW_MIN_) were calculated (13, 16).

### 2.3 Disease severity and functional outcome acquisition

#### 2.3.1 Questionnaire and hand function test

Participants completed a Health Assessment Questionnaire (HAQ) (21) and the Disabilities of Arm, Shoulder, and Hand (DASH) (22). Following the questionnaires, participants were administered the Jebsen-Taylor Hand Function Test (JTHFT) (23), which consists of seven tasks meant to evaluate hand functionality when performing day-to-day routine tasks (e.g., writing, lifting light and heavy weights, etc.)

#### 2.3.2 Ultrasound acquisition and processing

Ultrasound scans (Logiq S8, software version R2.2, GE HealthCare, Chicago, IL, United States) were performed by an ultrasound-trained rheumatologist (CP > 10 years). Both MCP2 and MCP3 joints of the dominant and non-dominant hand were evaluated for effusion/synovial hypertrophy and inflammation using power doppler. Based on these assessments, the rheumatologist chose the most affected hand to be used for the following HR-pQCT imaging analysis – to note, this chosen hand may not always be the participant’s dominant hand. Additionally, the MCP2 – MCP5 joints from the most affected hand were further evaluated for the presence of erosions. Effusion and synovial hypertrophy and inflammation grades were presented as the interquartile range (IQR) on a 0–3 grading scale. Grading was performed by two trained assessors (CP *>* 10 years and AI >3 years of experience).

### 2.4 Statistical analysis

Two-way ANOVAs for each MCP joint (MCP2 and MCP3) and for each joint space metric with diagnosis (RA, control) and sex (male, female) as between-subject factors were performed. Multiple linear regression analyses were performed to compare joint space parameters as a function of age, BMI, and JTHFT test times. To compare JTHFT (continuous variable) against ordinal ultrasound scores (i.e., effusion/synovial hypertrophy and power doppler), a Kendall rank correlation was utilized. Non-parametric, paired t-tests (Wilcoxon) were performed to compare clinical and demographic variables between RA and controls. Specifically, we compared matched hands between groups (i.e., dominant and non-dominant hands for JTHFT times).

While criteria for ultrasound grading (effusion/synovial hypertrophy and power doppler) have been developed they are dependent on the subjective assessment of the observed ultrasound signal. Thus, an intraclass correlation coefficient (ICC) between the two raters was performed. Specifically, we utilized a two-way random effects, single rater, absolute agreement exam (RStudio v2022.12.0 + 353). Classification of ICC values were as follows: < 0.05 = poor reliability, 0.5–0.75 = moderate reliability, 0.75–0.9 = good reliability, and > 0.9 = excellent reliability (24).

All statistical analysis were performed in Prism (v9.4.1, GraphPad, San Diego, CA, United States) unless otherwise noted. Statistical significance was noted if *p <* 0.05.

## 3 Results

### 3.1 Characteristics of the cohorts

From the initial 80 participants, *N* = 5 RA participants and their *N* = 5 matched controls were excluded from all analyses. The five RA participants were excluded due to a joint replacement (*N* = 1), presence of gout (*N* = 1), missed study appointment (*N* = 1), and asked for their data to be withdrawn from the study (*N* = 2). The demographic and clinical characteristics of the 35 RA patients and their 35 controls are shown in Table 1.

**Table 1:** Demographics of the healthy control (*N* = 35) and RA (*N* = 35) participants.

	Control (*N* = 35)	RA (*N* = 35)	*p*-value
Age	57.0 ± 15.0	57.4 ± 14.7	0.90
Female : Male (Female %)	27 : 8 (77%)	27 : 8 (77%)	N/A
Height (m)	1.57 ± 0.39	1.57 ± 0.39	0.97
Weight (kg)	72.4 ± 17.6	85.5 ± 21.8	0.008*
BMI (kg/m^2^)	26.2 ± 5.7	30.9 ± 6.7	0.003*
**Clinical parameters**			
Disease duration (years) (*N* = 33)	N/A	15.0 ± 10.1	N/A
28 Swollen joint count (*N* = 33)	N/A	1.7 ± 2.8	N/A
28 Tender joint count (*N* = 33)	N/A	3.0 ± 4.1	N/A
HAQ (0–3) (*N* = 35)	0.03 ± 0.1	0.86 ± 0.65	<0.001*
Patient global score (0–100) (*N* = 35)	1.8 ± 8.7	25.3 ± 30.5	<0.001*
AM stiffness (min) (*N* = 35)	1.1 ± 3.2	57.0 ± 153.4	0.04*
DAS28CRP (*N* = 33)	N/A	3.1 ± 1.3	N/A
DAS28ESR (*N* = 33)	N/A	3.1 ± 1.5	N/A
CDAI (*N* = 33) *Low CDAI* (> 2.8 and ≤ 10) *Mid CDAI* (> 10 and ≤ 22) *High CDAI* (> 22)	N/A N/A N/A N/A	10.3 ± 8.4 24 (73%) 4 (12%) 5 (15%)	N/A N/A N/A N/A
Medications *Corticosteroids* *DMARDs* *Biologic DMARD* *NSAIDs*	N/A N/A N/A N/A	2 (6%) 14 (42%) 19 (58%) 3 (9%)	N/A N/A N/A N/A
**Jebsen-Taylor hand function test times (s)**			
Most affected hand	46.3 ± 8.4	65.5 ± 25.5	< 0.001*
Contralateral hand	67.3 ± 14.0	66.1 ± 17.2	0.75
**Disabilities of the arm, shoulder, and hand (DASH)**			
Score	27.6 ± 5.7	56.0 ± 19.1	< 0.001*
Serological parameters			
RF (+: -) (*N* = 30)	N/A	23: 7	N/A
ACPA (+: -) (*N* = 14)	N/A	10: 4	N/A
ESR (*N* = 33)	N/A	14.9 ± 14.0	N/A
CRP (*N* = 33)	N/A	6.2 ± 7.7	N/A

Multiple parameters for the control were marked as N/A, as the data were not collected or relevant for healthy controls. All values are representative of *N* = 35 for each group of participants, unless noted otherwise, and are presented as mean ± standard deviation. *p*-values reflect results of a paired, non-parametric, *t*-test (Wilcoxon) comparing RA and control participants. Serological parameters were not available for all participants.

For the RA participants, the swollen and tender joint count, CDAI, and current medications were collected at their last visit to a rheumatologist, 18 ± 56 days relative to the imaging day. As blood work (ESR and CRP, contributing to DAS28 scores) was performed less frequently, these results were acquired 138 ± 236 days relative to the imaging day. Two RA participants were not registered within the Rheum4U database; thus, some of their metrics were not recorded (i.e., disease duration, swollen and tender joint count, blood work, and current medications). While some of the clinical and serological parameters were marked as N/A for the control participants, part of the inclusion criteria of the control participants required no hand injuries and no diagnosed conditions involving the hand. Thus, swollen and tender joint counts, disease duration, disease severity criteria (i.e., DAS28CRP/ESR and CDAI), medications, and serological parameters were not assessed for these participants.

Functional tests, questionnaires, and ultrasound grading revealed significant group-level differences between RA and controls. The RA group had greater pain and disability than their age-and sex-matched controls, resulting in significant differences between groups in patient global score (*p <* 0.001), morning stiffness (*p <* 0.001) and DASH scores (22) (*p <* 0.001). These self-reported metrics were also reflected in functional tests examining gross and fine motor skills, whereby Jebsen-Taylor hand function test (JTHFT) scores (23) were significantly poorer (*p <* 0.05) in the most affected hand for RA patients than the controls. With regards to ultrasound grading, scores for effusion/synovial hypertrophy and inflammation (i.e., positive power doppler signal) were significantly higher in the RA participants when compared to their age-and sex-matched controls in MCP2 and MCP3 of both hands (Table 2). The number of erosions present on ultrasound exam in MCP2 - MCP5 joints of the most affected hand was higher in RA than control participants (Table 2). The results of ICC revealed poor (effusion/synovial hypertrophy) to excellent (power doppler) reliability (Supplementary Table S1) with a higher consistency in the MCP2 joint, compared to MCP3.

**Table 2:** Results of ultrasound scores (effusion/synovial hypertrophy, power doppler, erosions) and comparisons between hand JTHFT times against ultrasound scores for both control and RA participants.

	Ultrasound scores			Ultrasound scores vs. JTHFT	
				*p*-value	
	Control	RA	*p*-value	Control	RA
**Effusion / synovial hypertrophy (0–3)**					
Most affected hand (MCP2) Contralateral hand (MCP2) Most affected hand (MCP3) Contralateral hand (MCP3)	1 [IQR 1–1] 1 [IQR 1–1] 1 [IQR 1–1] 1 [IQR 1–1]	1 [IQR 1–2.5] 1 [IQR 1–1.5] 1 [IQR 1–1] 1 [IQR 1–1]	< 0.001* 0.005* 0.02* 0.13	0.38 0.75 N/A 0.55	0.52 0.23 0.40 0.81
**Power doppler (0–3)**					
Most affected hand (MCP2) Contralateral hand (MCP2) Most affected hand (MCP3) Contralateral hand (MCP3)	1 [IQR 1–1] 1 [IQR 1–1] 1 [IQR 1–1] 1 [IQR 1–1]	1 [IQR 1–1] 1 [IQR 1–1] 1 [IQR 1–1.5] 1 [IQR 1–1]	0.008* 0.03* 0.004* 0.008*	0.39 0.34 N/A 0.55	0.98 0.97 0.11 0.83
**Erosions**					
MCP2 {# of People with Erosions} MCP3 {# of People with Erosions} MCP4 {# of People with Erosions} MCP5 {# of People with Erosions}	0 [IQR 0–0] {0} 0 [IQR 0–0] {1} 0 [IQR 0–0] {0} 0 [IQR 0–0] {0}	0 [IQR 0–1] {15} 0 [IQR 0–0] {7} 0 [IQR 0–0] {0} 0 [IQR 0–0] {3}			

For ultrasound scores, results are reported as the interquartile range, while *p* values of rank correlations were used for hand function vs. ultrasound scores. A significant correlation is noted if the *p* < 0.05. *p* values with a reported N/A were due to zero standard deviation amongst measurements. Significant values were bolded for emphasis.

### 3.2 Effects of diagnosis and sex on 3D JSW

**Figure 1:**
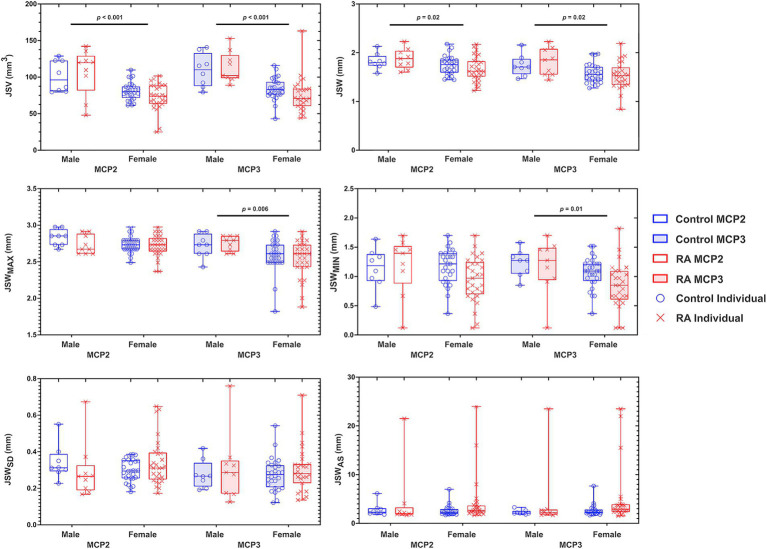
Graphs depicting the results of a two-way ANOVA comparing the effects of sex (male and female) and diagnosis (RA and control) on 3D joint space in the MCP2 and MCP3. Each individual data point for RA (X) and control (O) are presented within a bounding box, displaying the max and min value and mean (bar). Analyzed joint space metrics included the joint space volume (JSV), width (JSW), standard deviation (JSW_SD_), maximum width (JSW_MAX_), minimum width (JSW_MIN_), and joint space width asymmetry (JSW_AS_). To note, because JSW_AS_ is JSW_MAX_/JSW_MIN_, individuals with significant joint space narrowing can result in large JSWAS – demonstrating the large amount of individual variability within this metric. *N* = 70.

We observed no significant differences in joint space metrics as a function of diagnosis (i.e., RA or control) and no interactions between sex and diagnosis were noted (Figure 1). When examining the relationship between joint space changes as a function of disease duration and CDAI, as a longer disease duration did not imply worsening disease (i.e., CDAI), the sole significant association was found between disease duration and JSW_MAX_ (*p* = 0.04). No other correlations were noted for the remaining 3D joint space metrics. For sex, we found that females had significantly smaller JSV (MCP2, *p <* 0.001 and MCP3, *p <* 0.001); JSW (MCP2, *p =* 0.02 and MCP3, *p =* 0.02); JSW_MAX_ (MCP3, *p =* 0.006); and JSW_MIN_ (MCP3, *p =* 0.01) than males (Figure 1).

### 3.3 Age, BMI, and hand function test

There was a significant negative association between age and JSW_MAX_ in both MCP2 and MCP3 (*p =* 0.03). This association was affected by diagnosis (significant interaction between age and diagnosis; *p =* 0.001 and *p =* 0.04 for MCP2 and MCP3, respectively). For the remaining 3D joint space metrics (i.e., JSV, JSW, JSW_SD_, JSW_MAX_, JSW_MIN_, and JSW_AS_) we observed no significant association with age, diagnosis, nor an interaction effect. Additionally, we observed no significant associations with BMI and JTHFT for any of the 3D joint space metrics (Table 3).

**Table 3:** Results of multiple linear regression analyses of 3D joint space metrics as a function of age, BMI, and JTHFT for both MCP2 and MCP3 joints.

	Age				BMI				JTHFT			
	MCP2		MCP3		MCP2		MCP3		MCP3		MCP3	
	*R* ^2^	*p-*value	*R* ^2^	*p-*value	*R* ^2^	*p-*value	*R* ^2^	*p-*value	*R* ^2^	*p-*value	*R* ^2^	*p-*value
JSV	0.08	0.14	0.04	0.24	0.01	0.82	0.02	0.60	0.03	0.54	0.03	0.90
JSW	0.01	0.65	0.03	0.44	0.05	0.15	0.07	0.05	0.02	0.64	0.06	0.59
JSW_SD_	0.03	0.29	0.06	0.08	0.03	0.76	0.04	0.80	0.02	0.58	0.02	0.71
JSW_MAX_	**0.12**	0.03*	**0.08**	0.03*	0.08	0.15	0.12	0.09	0.06	0.81	0.09	0.84
JSW_MIN_	0.06	0.37	0.09	0.53	0.04	0.51	0.08	0.40	0.05	0.58	0.10	0.66
JSW_AS_	0.06	0.77	0.07	0.72	0.08	0.89	0.08	0.98	0.06	0.79	0.10	0.94

Displayed are the *R*^2^ values with their associated *p* values. *A significant correlation was noted if *p* < 0.05. Significant values were bolded for emphasis.

## 4 Discussion

The results of our study demonstrated that HR-pQCT-derived 3D JSW metrics did not differ between RA patients and an age-and sex-matched control group despite differences in disease severity and functional outcomes. As expected, there was a significant association between some metrics, in particular JSW_MAX_, with age and sex. Only the association between JSW_MAX_ and age depended on diagnosis.

Larger JSV, JSW, and JSW_MAX_ in males than females were consistent with previous findings, and the observation that these metrics are related to overall joint size more than joint degeneration (15). The finding that minimum JSW (JSW_MIN_) was smaller in females than males in MCP3 requires further investigation as this was not consistent with results in previous studies using only RA patients, and may reflect the relatively small sample of males in the current study.

The absence of significant associations between JSW metrics and age, with the exception of JSW_MAX_, suggests that MCP2 and MCP3 are not significantly affected by age-related degeneration. Hand osteoarthritis can cause joint space narrowing, however the distal interphalangeal, proximal interphalangeal and first carpometacarpal joints are more commonly affected than the MCP joints (25). Nonetheless, as cortical bone interruptions, indicative of erosive damage, increase with age (26) we recommend considering age as an important variable in HR-pQCT studies in RA.

Obesity can cause systemic inflammation, which is known to be associated with an increased incidence of RA (27, 28). Despite its association with RA, some studies have shown a negative correlation between joint space and obesity (29), while other studies have shown a protective effect (30, 31). In our study, HR-pQCT acquired 3D joint space metrics were not associated with BMI, thus (a) higher BMI may have had a protective role or (b) our metrics were not sensitive enough to detect the small changes that may be associated with BMI.

Overall, our findings suggest that HR-pQCT-derived 3D JSW metrics cannot identify significant group-level differences in 3D JSW metrics between RA patients and age-and sex-matched controls. Further research may be required to determine whether this finding reflects the absence of a true difference between groups or an inability to detect true differences with HR-pQCT. Lack of group-level differences may be attributed in part overall well-controlled disease activity within this group of RA participants (Supplementary Figure S1). In the future, with a larger sample size, age-, and sex-, dependent MCP Z-scores could be calculated. Similar work has been previously shown in the proximal interphalangeal joint – which have shown age-and sex-dependent joint space decreases (32, 33). Nonetheless, our measures of disease activity and function did demonstrate that, on average, the control group had healthier joints. Further research should investigate whether HR-pQCT assessed JSW metrics can be used for the purposes of clinical trials to sensitively detect changes over time.

## Data availability statement

The raw data supporting the conclusions of this article will be made available by the authors, without undue reservation.

## Ethics statement

The studies involving humans were approved by the Conjoint Health Research Ethics Board (CHREB), University of Calgary. The studies were conducted in accordance with the local legislation and institutional requirements. The participants provided their written informed consent to participate in this study.

## Author contributions

JT: Data curation, Formal analysis, Investigation, Methodology, Project administration, Visualization,Writing – original draft, Writing – review & editing. DC: Data curation, Writing – review & editing. PS: Data curation, Investigation, Methodology, Writing – review & editing. CEHB: Investigation, Methodology, Resources, Writing – review & editing. GH: Investigation, Methodology, Resources, Writing – review & editing. ChB: Conceptualization, Writing – review & editing. CP: Data curation, Investigation, Methodology, Writing – review & editing. AI: Data curation, Investigation, Methodology, Writing – review & editing. DM: Conceptualization, Writing – review & editing. SM: Funding acquisition, Methodology, Project administration, Supervision, Writing – original draft.
