# Supplementary material for: Evaluating high-resolution computed tomography derived 3-D joint space metrics of the metacarpophalangeal joints between rheumatoid arthritis and age- and sex-matched control participants
Source: Front Med (Lausanne). 2024 May 9;11:1387532. doi: 10.3389/fmed.2024.1387532 (PMC11112086; doi:10.3389/fmed.2024.1387532)
Supplement: Supplementary file 1 [file Data_Sheet_1.docx]

Supplementary Material

**Evaluating High-Resolution Computed Tomography Derived 3-D Joint Space Metrics of the Metacarpophalangeal Joints Between Rheumatoid Arthritis and Age- and Sex-Matched Control Participants**

**Justin J. Tse^1,2^, Dani Contreras^1,3^, Peter Salat^2^, Claire E.H. Barber^1,4^, Glen S. Hazlewood^1,4^, Cheryl Barnabe^4^, Chris Penney^4^, Ahmed Ibrahem^4^, Dianne Mosher^4^, and Sarah L. Manske^1,2*^**

*** Correspondence:** Sarah L. Manske: smanske@ucalgary.ca


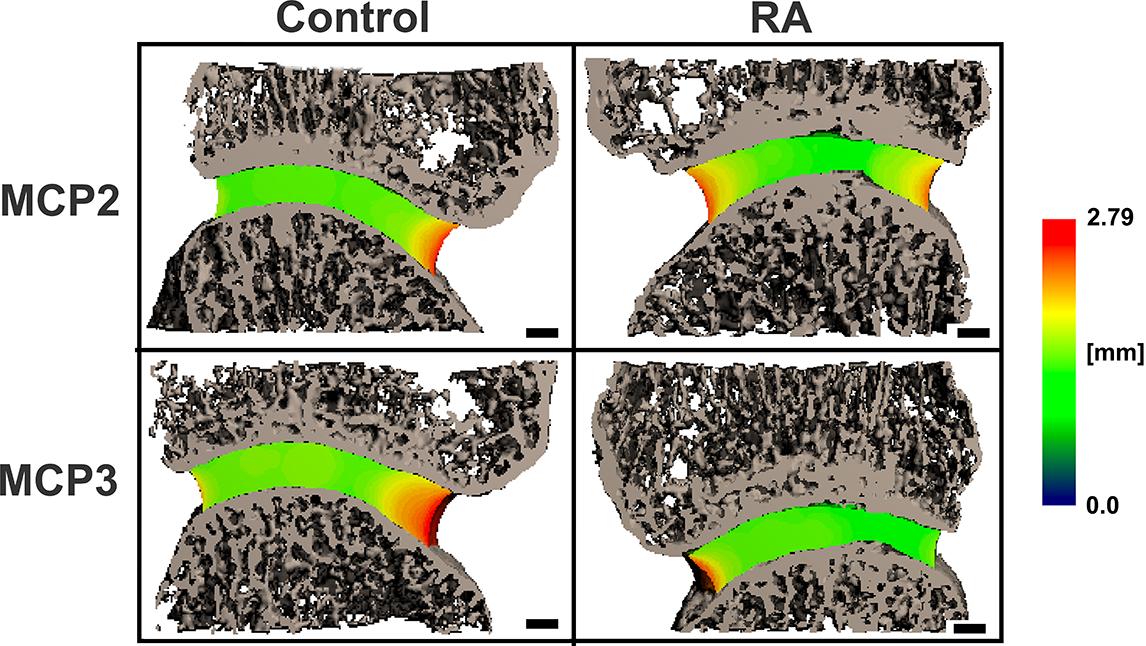


**Supplementary Figure 1.** Image depicting the joint space width (JSW) of the MCP2 and MCP3 joints from a single representative participant from the control and RA group. The participants were chosen based on the proximity of their JSW to the median JSW of each group. Black scalebar = 1 mm

Supplementary Table 1. Results of a two-way random effects single rater absolute agreement intraclass correlation coefficient (ICC) tests between two raters for ultrasound scores for the RA and control participants. The ICC values and their 95% confidence intervals are presented.

|  | **ICC** | **ICC 95% Confidence Interval** |
| --- | --- | --- |
| **Effusion / Synovial Hypertrophy** |  |  |
| Right MCP2 | 0.313 | -0.086 < ICC < 0.609 |
| Right MCP3 | 0.520 | 0.08 < ICC < 0.748 |
| Left MCP2 | 0.519 | 0.077 < ICC < 0.748 |
| Left MCP3 | 0.292 | -0.035 < ICC < 0.548 |
| **Power Doppler (PD)** |  |  |
| Right MCP2 | 0.929 | 0.885 < ICC < 0.957 |
| Right MCP3 | 0.396 | 0.162 < ICC < 0.589 |
| Left MCP2 | 0.828 | 0.730 < ICC < 0.893 |
| Left MCP3 | 0.788 | 0.671 < ICC < 0.867 |
